# Accuracy of the Simplified Thylstrup & Fejerskov Index in Rural Communities with Endemic Fluorosis

**DOI:** 10.3390/ijerph7030927

**Published:** 2010-03-09

**Authors:** Ana Karoline Adelário, Lívia F Vilas-Novas, Lia S Castilho, Andréa Maria D Vargas, Efigênia F Ferreira, Mauro Henrique N. G Abreu

**Affiliations:** 1 Department of Community and Preventive Dentistry, Universidade Federal de Minas Gerais, Avenida Antônio Carlos, 6627–31270.901 sala 3304, Belo Horizonte, MG, Brazil; E-Mails: anakaroline2004@hotmail.com (A.K.A.); liviavilasnovas@hotmail.com (L.F.V.-N.); vargasnt@task.com.br (A.M.D.V.); efigeniaf@gmail.com (E.F.F); 2 Department of Operative Dentistry, Universidade Federal de Minas Gerais, Avenida Antônio Carlos, 6627–31270.901, Belo Horizonte, MG, Brazil; E-Mail: liacastilho@ig.com.br

**Keywords:** accuracy, dental fluorosis, Thylstrup & Fejerskov Index

## Abstract

The aim of the present study was to compare the values of the Thylstrup & Fejerskov Index (TF index) for the determination of the prevalence of dental fluorosis using either all teeth (gold standard) or six upper anterior teeth (simplified TF index). The sample was made up of 396 individuals aged six to 22 years from three Brazilian cities with endemic fluorosis caused by the ingestion of water with high fluoride concentration. The prevalence of dental fluorosis was evaluated by a single trained examiner with excellent intraexaminer agreement (kappa = 0.95). Intraexaminer reproducibilities were calculated at tooth level. Sensitivity, specificity, positive and negative predictive values of the simplified TF compared to gold standard were 90.6 (95%CI: 86.6 to 93.6), 100 (95%CI: 95.3 to 100), 100 (95%CI: 98.3 to 100) and 77.5 (95%CI: 69.8 to 83.5), respectively. The ROC value was 0.953 (95%CI: 0.933 to 0.973). The simplified TF index proved suitable for determining the prevalence of dental fluorosis in regions with endemic fluorosis caused by the ingestion of water with high concentrations of fluoride.

## 1. Introduction

Dental fluorosis stems from the exposure of the tooth germ to high concentrations of fluoride during the formative period. Clinically, dental fluorosis is characterized by a mostly symmetrical distribution in homologous teeth. Milder forms exhibit thin, opaque lines often crossing the entire surface of the enamel. In more severe cases, the dental structure acquires a brownish coloration resulting from the porous enamel. The distribution and severity of this condition are affected by the amount of fluoride ingested, individual susceptibility, age and exposure time [1].

The most often employed measures for the determination of dental fluorosis are the Dean Index (1942) and the Thylstrup & Fejerskov (TF) index (1978). The TF index classifies dental fluorosis based on clinical appearance, with scores ranging from 0 to 9, thereby allowing the determination of the mildest to most severe forms of dental fluorosis. At a score of 0, the enamel represents the normal translucency. Increasing values of the ordinal scale of the index denote an increase in the severity of fluorosis: Scores 1 to 4 denote increasing degrees of opacity with no loss of outermost enamel. Scores of 5 or more denote increasing degrees of loss of outermost enamel [1,2]. The TF index is very useful in regions with endemic fluorosis caused by the ingestion of water with a fluoride concentration above recommended levels.

In endemic dental fluorosis, a significant portion of the local population exhibits a moderate to severe degree of this condition. Under such circumstances, dental fluorosis is considered a public health problem, as the functional and aesthetic alterations have an effect on self esteem and inclusion in the job market. Moreover, the etiology is known and prevention is feasible [3–5]. A number of studies have demonstrated the association between endemic dental fluorosis and the ingestion of groundwater with high fluoride concentrations [6–8]. In Brazil, endemic dental fluorosis has been reported in rural communities of the municipalities of São Francisco, Verdelândia and São João das Missões in the northern portion of the state of Minas Gerais. The fluorosis endemic in these communities is directly related to the ingestion of groundwater with a fluoride content ranging from 1.17 to 4.6 mg/L. These values are well above the optimal content (0.7 mg/L) defined by the Brazilian Ministry of Health [9].

The diagnosis of dental fluorosis using the TF index is performed by examining all the teeth. In epidemiological studies, the examination of all teeth requires more time. Medina-Solis *et al.* (2008) assessed the prevalence of dental fluorosis using the modified Dean Index on adolescents in the city of Tula de Allende (Mexico). The city had a fluoride concentration level between 1.38 and 3.07 mg/L and had an 81.7% prevalence of fluorosis. Mild and very mild fluorosis were diagnosed in 51.1%. Moderate and severe dental fluorosis were diagnosed in 10.5% and 19.1%, respectively. The authors found high specificity and positive predictive values (100%) when comparing the diagnosis using six teeth (upper incisors and canines) and that using 28 teeth. Sensitivity and negative predictive values were 71.8% and 44.3%, respectively [10]. The evaluation of the accuracy of simplified fluorosis measures is important; as such measures may allow a more rapid assessment of the epidemiology of dental fluorosis in communities exposed to water supplies with high fluoride content. Despite the results described by Medina-Solis *et al.* [10], the accuracy of the simplified TF index has not yet been assessed.

The aim of the present study was to determine the accuracy of the simplified TF index (upper anterior teeth) in comparison to the full mouth TF index and evaluate the influence of gender and age.

## 2. Results and Discussion

A total of 396 individuals between six and 22 years of age, residents from the rural districts of São Francisco, São João das Missões and Verdelândia in the state of Minas Gerais, Brazil, participated in the present study. The mean number of fully erupted teeth were 12.2 (±3.2), 21.6 (±5.8), 27.7 (±1.3) for individuals aged 6−9, 10−12 and 13−22 years, respectively. The following frequencies, at the individual level, were found for each score of the full TF index: 0 (24.5%), 1 (11.1%), 2 (9.1%), 3 (8.6%), 4 (7.1%), 5 (11.6%), 6 (9.3%), 7 (10.1%), 8 (6.1%), 9 (2.5%). The following frequencies, at the individual level, were found for each score of simplified TF index: 0 (31.6%), 1 (11.9%), 2 (10.4%), 3 (9.6%), 4 (8.6%), 5 (14.6%), 6 (5.6%), 7 (4.0%), 8 (3.2%), 9 (0.5%). Table 1 displays the prevalence of dental fluorosis according to age and gender. The prevalence of fluorosis determined by the full mouth TF index was 75.5%, whereas the simplified TF index determined a prevalence of 68.4%.

Table 2 displays the values of the simplified TF index in comparison to the gold standard (full mouth TF index) according to gender and age. Overall sensitivity was 90.6% (95%CI: 86.6 to 93.6). Overall specificity was 100% (95%CI: 95.3 to 100.0). The positive predictive value was 100% (95%CI: 98.3 to 100.0). The negative predictive value was 77.5% (95%CI: 69.8 to 83.5). The area under the ROC curve was 0.953 (95%CI: 0.933 to 0.973). Sensitivity values were above 85% and specificity values were 100% for all ages and both genders. Subgroups of ROC curve values were high, ranging from 0.926 to 0.981. All positive predictive values were 100% and negative predictive values ranged from 68.8% to 89.5%.

Table 3 displays the prevalence of dental fluorosis according to age and gender when using the TF ≥ 3 cutoff point. The TF index determined a 55.3% prevalence of severe fluorosis, whereas the simplified TF index determined a prevalence of 46.2%. Differences in the prevalence of severe dental fluorosis between both indices ranged from 4.8% to 13.7%. These differences were 8.2% (6–9 years old), 13.7% (10–12 years old) and 4.8% (13–22 years).

Table 4 displays the values obtained with the TF ≥ 3 cutoff point using the simplified TF index in comparison to the gold standard (full mouth TF index), according to gender and age. Overall sensitivity was 83.6% (95%CI: 77.8 to 88.1). Overall specificity and positive predictive values were 100% (95%CI: 97.4 to 100.0). The negative predictive value was 83.1% (95%CI: 78.0 to 87.2). The area under the ROC curve was 0.918 (95%CI: 0.888 to 0.948). Sensitivity values were above 76% and specificity values were 100% for all ages and both genders. ROC curve values were high, ranging from 0.882 to 0.963. All positive predictive values were 100% and negative predictive values ranged from 77.3% to 91.6%.

Table 5 displays the prevalence of dental fluorosis according to age and gender when using the cutoff point of TF index ≥ 5. The TF index determined a 39.6% prevalence of severe fluorosis, whereas the simplified TF index determined a prevalence of 28.0%. Differences in the prevalence of severe dental fluorosis between both indices ranged from 8.8% to 14.6%. These differences were 8.8% (6−9 years old), 14.6% (10−12 years old) and 12.3% (13−22 years).

Table 6 displays the values obtained with the cutoff point of TF index ≥ 5 using the simplified TF index in comparison to the gold standard (full mouth TF index), according to gender and age. Overall sensitivity was 70.7% (95%CI: 62.8 to 77.5). Overall specificity and positive predictive values 100% (95%CI: 100.0 to 100.0). Negative predictive value was 83.9% (95%CI: 80.4 to 87.1). The area under the ROC curve was 0.854 (95%CI: 0.809 to 0.898). Sensitivity values were above 64% and specificity values were 100% for all ages and both genders. ROC curve values were high, ranging from 0.821 to 0.895. All positive predictive values were 100% and negative predictive values ranged from 81.0% to 87.9%.

### Discussion

The epidemiological diagnosis of health conditions involves the use of indicators. Simplicity and validity are important requirements for a good public health indicator. These measures must also be reproducible and consensually interpretable. The use of an indicator with such characteristics allows a valid and reliable diagnosis at an affordable cost and allows the evaluation of health conditions that are interrelated with the environment [11].

A number of epidemiological indices have been developed for the measurement of dental fluorosis. Those proposed by Dean (1942) and Thylstrup & Fejerskov (1978) are the most often employed for the description of the clinical aspects of fluorosis [2]. A number of aspects limit the use of the Dean Index, such as its lower sensitivity in comparison to the TF index and its inability to clearly describe the more severe forms of this condition. According to Fejerskov *et al.* [1] and Thylstrup & Fejerskov [12], the TF index was drafted with the purpose of modifying and improving the Dean Index and is more indicated for use on populations that have a high prevalence of dental fluorosis and excessive exposure to fluoride. The benefits of the TF index include its accuracy, sensitivity and capability of characterizing dental fluorosis in populations that exhibit the entire gamut of this condition. Moreover, the TF index can correlate clinical aspects with histological aspects of the affected enamel, which are characteristics of considerable importance to clinicians and epidemiologists [1,2,12]. Thus, the TF index is more useful in regions in which the determinants of fluorosis are associated to the ingestion of water with a high fluoride concentration, such as the region investigated in the present study.

Medina-Solis *et al.* (2008) examined adolescent residents in a municipality in Mexico using the modified Dean Index for the determination of dental fluorosis. In this municipality, the analysis of the public drinking water revealed a fluoride concentration ranging from 1.38 mg/L to 3.07 mg/L. The difference in the prevalence of dental fluorosis when determined using either 28 or only six teeth was 23% [10], which is greater than that found in the present study (7.1%).

As demonstrated in a previous study [12], the prevalence and severity of dental fluorosis is directly related to the amount of fluoride ingested, age at the time of exposure and duration of exposure. The highest prevalence of severe fluorosis was observed among individuals between 13 and 22 years of age. The fewer fully erupted teeth among younger children may explain this finding. Moreover, two rural communities in the municipality of São Francisco stopped consuming water with high fluoride levels in 1995 and 1996. Children who were 6–9 years old (n = 36) in 2002 (year of the epidemiological survey) in these two communities had had fewer years of exposure to fluoride.

There is a progressive reduction in sensitivity and accuracy when higher cutoff points are used. Thus, TF 3 cutoff point achieved intermediate values whereas TF 5 cutoff point achieved the lowest sensitivity and accuracy values. The lack of an evaluation of the second molars premolars seems to affect the accuracy of the simplified TF index more when assessing the severity of fluorosis (TF index ≥ 3 and 5) than when assessing the overall prevalence of the condition. On the other hand, the negative predictive values increased when using TF 3 and 5 cutoff points. The reduction of the prevalence of dental fluorosis, considering these cutoff points, influenced the increasing of negative predictive values. The sensitivity value (70.7; 95%CI: 62.8 to 77.5) demonstrates that the simplified TF index has difficulty in correctly diagnosing individuals with TF ≥ 5. All PPV and specificity values were 100% by definition, since the simplified index is subset of full index.

The reduction in time spent on clinical exams and the elimination of costs related to dental equipment and materials are some of the benefits of the simplified TF index for the determination of dental fluorosis. Further studies are needed to evaluate the time spent applying both indexes. In the organization of the present epidemiological survey, many exams were performed in a group setting rather than a home-based setting, which also helps to save resources.Thus, this simplified version may be useful for the determination of the prevalence of dental fluorosis (although with limitation on severity evaluation) in regions with endemic fluorosis associated to environmental characteristics such as the ingestion of water with high fluoride concentrations.

It should be stressed that the evaluation of overall prevalence is of lesser public health importance than the evaluation of higher scores of fluorosis. TF scores equal to or greater than three (with remarkable opacities) and equal to or greater than five (with loss of outermost enamel) have an impact on quality of life. A valid, reliable epidemiological diagnosis may allow interdisciplinary policies addressing this public health problem to be implemented sooner.

Due to the impossibility of using a random sample, a convenience sample was used. Another limitation was the inclusion of children under 12 years of age, once only 23.4% of this age group had all six upper anterior teeth erupted. The majority of our sample had 12 years or less, so we decided to include this age on TF simplification. Considering that TF simplification for children without some of upper anterior teeth have limitations, we have analyzed TF simplification by age group.

This proposal of a simplified TF index is unique in the public health literature. Further studies on the simplification of the TF index should be carried out in regions with endemic fluorosis as well as in regions with adequate concentrations of fluoride in the drinking water.

## 3. Experimental Section

The Human Research Ethics Committee of the Federal University of Minas Gerais (Brazil) approved the protocol for this study. An explanation was given to the parents/guardians regarding the objectives and methods of the study; anonymity of data was ensured for the subjects; all doubts were clarified before initiating the study; and parents/guardians of the children examined signed terms of informed consent.

Oral health epidemiological surveys were carried out in the municipalities of São Francisco, Verdelândia and São João das Missões in the northern portion of the state of Minas Gerais, Brazil. São Francisco has an estimated population of 53,000 inhabitants, with a high poverty index and compromised quality of living and health [4,9]. The climate is semi-arid, with a mean annual temperature of 26º C and annual rainfall of 1132.9 mm, concentrated in four months of the year. The water supply in the rural districts has been compromised by drought and the loss of wetlands. The municipalities of Verdelândia and São João das Missões have estimated populations of 8000 and 11,000 inhabitants, respectively, and have similar climatic and socioeconomic aspects as those found in São Francisco [4]. In 1979, wells were sunk to access groundwater for the public supply and high levels of fluoride were detected after a number of years [9]. The same water system (well water) was available for all in a community. A sampling campaign of deep groundwater in the rural communities of interest was carried out concomitantly to the epidemiological survey (2002, 2005 and 2007), measuring the fluoride concentration in the laboratory. Due to the low concentration of aluminum and low turbidity of the groundwater of the region, the fluoride analytical method adopted was SPADNS photometry using a portable digital Hach colorimeter with precision of ±0.02 mg/L and Hach Spadns reagent. Measurements were made in duplicate for certification of analytical results: six samples were sent to two different laboratories for comparison of fluoride analysis, both using SPADNS method. Analytical, water collection and water preservation procedures were carried out in compliance with the Standard Methods for the Examination of Water and Wastewater, 20th edition [13]. An analysis of the well water revealed a fluoride concentration ranging from 1.17 to 4.6, which is higher than the limit recommended by the Brazilian Ministry of Health.

Epidemiological surveys were carried out in the rural communities of these three municipalities in 2002, 2005 and 2007. In 2002, residents in the rural communities of Mocambo (3.2 mg/L of fluoride), Vaqueta (3.0 mg/L of fluoride) and Novo Horizonte (3.9 mg/L of fluoride) in the municipality of São Francisco were surveyed. In 2005, the Amargoso (4.6 mg/L of fluoride) district of Verdelândia and rural districts of São João das Missões (1.17 mg/L of fluoride) were surveyed. In 2007, the communities of Barreiro dos Anjicos (2.2 mg/L of fluoride), Brejo dos Anjicos (2.6 mg/L of fluoride) and Furado Grande (1.4 mg/L of fluoride) in the municipality of São Francisco were surveyed.

The population selected for the present study was made up of individuals between six and 22 years of age. The age of six years was chosen due to the beginning of the change in dentition. The upper limit age of 22 years was chosen because individuals above this age may nothave been exposed to the excessive ingestion of fluoride from well water during the formation of their dental enamel. There is little information on the target population—children that lived in one of eight rural communities from three municipalities. The overall 6-to-22-year-old population was estimated at 22,175 in the whole of São Francisco, 3077 in the whole of Verdelândia and 1022 in the whole of São João das Missões. However, this information is less useful, since we do not know the number of people that lived in each rural community. Considering this limitation, our convenience sample was achieved with the help of representatives of the respective city halls and community leaders. The latter discussed and explained the objectives of our study in each rural community and scheduled the visits for data collection. Most of the subjects were residents in the countryside at places very far from each other. On the previously scheduled day, fireworks were used to call the population for the exams. All individuals who appeared at the predetermined locations and gave informed consent were examined. Individuals missing all their permanent upper anterior teeth were excluded from the study. The sample size was calculated considering an 80% prevalence of fluorosis, with a 95% confidence level and a margin of error of 4%, which determined the need for 384 individuals. A total of 396 individuals with at least one permanent upper anterior tooth fully erupted were examined.

The clinical examinations were carried out by a single, previously trained examiner with excellent intraexaminer agreement (kappa = 0.95) at schools and healthcare posts under natural light, with prior plaque removal with a toothbrush. Intraexaminer reproducibilities were calculated at tooth level. All teeth were dried with gauze for the exam. No dental instruments (mirror, explorer) were used. The TF index was used for the diagnosis of fluorosis, examining all surfaces of all teeth [4], with each tooth scored based on the most affected surface. Scores ranged from 0 (absence of fluorosis) to 9 (severe fluorosis). The enamel represents the normal translucency in zero score. Increasing values of the ordinal scale of the index denote an increase in the severity of fluorosis. Scores 1 to 4 denote increasing degrees of opacity with no loss of outermost enamel. Scores of 5 or more denote increasing degrees of loss of outermost enamel [1,2]. No individual wore an orthodontic appliance. Teeth with dental restorations (n = 2) were not examined. Dental fluorosis diagnosis was based on a pattern of white opacities, with a diffuse distribution over the surface of varying intensity and other characteristics. The opacities can vary from minor white striations and lines to small or extensive area of opaque enamel. Loss of outermost enamel (pits) and staining of enamel were also considered. Homologous teeth are affected, but not all homologous teeth are identically affected [1,14].

The databank was constructed using the Statistical Package for the Social Sciences (SPSS version 17.0 for Windows®). The frequency and distribution of dental fluorosis were determined for age and gender. The following were calculated: sensitivity (capability of the index to identify the disease among individuals who are actually affected); specificity (capability of the index to discard the disease among individuals who are not affected); positive predictive value (proportion of individuals who are actually affected and tested positive for the disease); negative predictive value (proportion of individuals who are not affected and tested negative for the disease); and area under the receiver operating characteristic (ROC) curve. The TF index was used as the gold standard, which was based on the scores recorded for all teeth in the oral cavity. Analysis was performed considering gender, age and two cutoff points for dental fluorosis. The first cutoff point was the presence or absence of any degree of the condition (prevalence). The second and third cutoff points were TF ≥ 3 and TF ≥ 5 (condition in which there is loss of the dental structure) for the determination of prevalence of severe fluorosis. 95% confidence intervals were calculated.

## 4. Conclusions

The present proposal for a simplified TF index for the determination of the prevalence of dental fluorosis achieves a result close to that of the full mouth evaluation. The simplified TF index, especially when using TF ≥ 3 cutoff point, proved suitable for determining dental fluorosis in regions with endemic fluorosis caused by the ingestion of water with high concentrations of fluoride.

## Figures and Tables

**Table 1.** Prevalence of dental fluorosis according to age and gender using the full mouth TF index and the simplified TF index, rural communities, Brazil.

Variable	TF index	Simplified TF index	Difference
**Age**			
6–9 years (n = 159)	78.6%	71.7%	6.9%
10–12 years (n = 131)	72.5%	61.8%	10.7%
13–22 years (n = 106)	74.5%	71.7%	2.8%
Gender*			
Female (n = 194)	72.2%	68.0%	4.2%
Male (n = 201)	78.6%	70.6%	8.0%
**Total** (n = 396)	75.5%	68.4%	7.1%

* Data missing for one individual.

**Table 2.** Sensitivity, specificity, accuracy, positive and negative predictive values achieved using the simplified TF index to determine the presence or absence of any degree of dental fluorosis when compared to the gold standard (full mouth TF index), rural communities, Brazil.

**Variable**	Sensitivity (95%CI)	Specificity (95%CI)	ROC Area (95%CI)	PPV§ (95%CI)	NPV§ (95%CI)
**Age**					
6–9 years (n = 159)	91.2 (84.4–95.3)	100.0 (87.4–100.0)	0.956 (0.926–0.986)	100.0 (95.4–100.0)	78.7 (64.5–87.3)
10–12 years (n = 131)	85.3 (76.2–91.4)	100.0 (88.0–100.0)	0.926 (0.882–0.971)	100.0 (95.1–100.0)	68.8 (54.5–79.1)
13–22 years (n = 106)	96.2 (88.5–99.0)	100.0 (84.5–100.0)	0.981 (0–1.0)	100.0 (94.6–100.0)	89.5 (70.5–97.0)
Gender*					
Female (n = 194)	91.4 (85.2–95.3)	100.0 (91.7–100.0)	0.957 (0.929–0.985)	100.0 (96.9–100.0)	79.1 (66.8–87.3)
Male (n = 201)	89.9 (83.8–93.9)	100.0 (89.8–100.0)	0.949 (0.920–0.978)	100.0 (96.2–100.0)	76.3 (64.3–84.2)
**Total** (n = 396)	90.6 (86.6–93.6)	100.0 (95.3–100.0)	0.953 (0.933–0.973)	100.0 (98.3–100.0)	77.5 (69.8–83.5)

PPV: Positive predictive value; NPV: negative predictive value; CI: Confidence interval;

* data missing for one individual;

§ prevalence of dental fluorosis = 75.5% (See Table 1).

**Table 3.** Prevalence of severe fluorosis (TF index ≥ 3) according to age and gender using the full mouth TF index and simplified TF index, rural communities, Brazil.

Variable	TF index	Simplified TF index	Difference
**Age**			
6–9 years (n = 159)	47.2%	39.0%	8.2%
10–12 years (n = 131)	58.0%	44.3%	13.7%
13–22 years (n = 106)	64.2%	59.4%	4.8%
Gender*			
Female (n = 194)	52.1%	42.3%	9.8%
Male (n = 201)	58.2%	49.8%	8.4%
**Total** (n = 396)	55.3%	46.2%	9.1%

* Data missing for one individual.

**Table 4.** Sensitivity, specificity, accuracy, positive and negative predictive values achieved using the simplified TF index to determine the prevalence of severe dental fluorosis (TF ≥ 3) when compared to the gold standard (full mouth TF index), rural communities, Brazil.

Variable	Sensitivity (95%CI)	Specificity (95%CI)	ROC Area (95%CI)	PPV^§^ (95%CI)	NPV^§^ (95%CI)
**Age**					
6–9 years (n = 159)	82.7 (50.4–80.0)	100.0 (94.6–100.0)	0.913 (0.861–0.965)	100.0 (94.3–100.0)	82.4 (73.1–89.1)
10–12 years (n = 131)	76.3 (64.9–85.0)	100.0 (91.9–100.0)	0.882 (0.821–0.942)	100.0 (90.8–100.0)	77.3 (67.9–84.3)
13–22 years (n = 106)	92.6 (83.0–97.3)	100.0 (88.6–100.0)	0.963 (0.926–1.0)	100.0 (90.0–100.0)	91.6 (80.8–96.8)
Gender*					
Female (n = 194)	81.2 (71.9–88.0)	100.0 (95.1–100.0)	0.906 (0.859–0.953)	100.0 (94.8–100.0)	81.1 (73.2–87.1)
Male (n = 201)	85.5 (77.5–91.1)	100.0 (94.6–100.0)	0.927 (0.889–0.966)	100.0 (94.7–100.0)	84.8 (77.3–90.1)
**Total** (n = 396)	83.6 (77.8–88.1)	100.0 (97.4–100.0)	0.918 (0.888–0.948)	100.0 (97.4–100.0)	83.1 (78.0–87.2)

PPV: Positive predictive value; NPV: negative predictive value; CI: Confidence interval;

* data missing for one individual;

§ prevalence of dental fluorosis (TF ≥ 3) = 55.3% (See Table 3).

**Table 5.** Prevalence of severe fluorosis (TF index ≥ 5) according to age and gender using the full mouth TF index and simplified TF index, rural communities, Brazil.

Variable	TF index	Simplified TF index	Difference
**Age**			
6–9 years (n = 159)	26.4%	17.6%	8.8%
10–12 years (n = 131)	40.5%	25.9%	14.6%
13–22 years (n = 106)	58.5%	46.2%	12.3%
Gender*			
Female (n = 194)	38.6%	27.8%	10.8%
Male (n = 201)	40.8%	28.3%	12.5%
**Total** (n = 396)	39.6%	28.0%	11.6%

* Data missing for one individual.

**Table 6.** Sensitivity, specificity, accuracy, positive and negative predictive values achieved using the simplified TF index to determine the prevalence of severe dental fluorosis (TF ≥ 5) when compared to the gold standard (full mouth TF index), rural communities, Brazil.

Variable	Sensitivity (95%CI)	Specificity (95%CI)	ROC Area (95%CI)	PPV^§^ (95%CI)	NPV^§^ (95%CI)
Age					
6–9 years (n = 159)	66.7 (50.4–80.0)	100.0 (96.0–100.0)	0.833 (0.744–0.923)	100.0 (95.4–100.0)	82.1 (74.7–88.4)
10–12 years (n = 131)	64.2 (49.7–76.5)	100.0 (94.2–100.0)	0.821 (0.738–0.904)	100.0 (94.1–100.0)	81.0 (74.1–86.6)
13–22 years (n = 106)	79.0 (66.5–87.9)	100.0 (81.3–100.0)	0.895 (0.832–0.959)	100.0 (81.3–100.0)	87.9 (80.4–92.7)
Gender*					
Female (n = 194)	72.0 (60.3–81.5)	100.0 (96.1–100.0)	0.860 (0.797–0.923)	100.0 (96.6–100.0)	84.5 (78.7–89.2)
Male (n = 201)	69.5 (58.2–78.9)	100.0 (96.1–100.0)	0.848 (0.785–0.910)	100.0 (96.5–100.0)	83.3 (77.8–87.8)
**Total** (n = 396)	70.7 (62.8–77.5)	100.0 (100.0–100.0)	0.854 (0.809–0.898)	100.0 (100.0–100.0)	83.9 (80.4–87.1)

PPV: Positive predictive value; NPV: negative predictive value; CI: Confidence interval;

* data missing for one individual;

§ prevalence of severe dental fluorosis = 39.6% (See Table 5).
